# Fitness-for-purpose of the CanMEDS competencies for workplace-based assessment in General Practitioner’s Training: a Delphi study

**DOI:** 10.1186/s12909-023-04207-2

**Published:** 2023-04-01

**Authors:** Vasiliki Andreou, Sanne Peters, Jan Eggermont, Mieke Embo, Nele R. Michels, Birgitte Schoenmakers

**Affiliations:** 1 Academic Centre for General Practice, Department of Public Health and Primary Care, KU Leuven, Box 7001, Kapucijnenvoer 7, Leuven, 3000 Belgium; 2 School of Health Sciences, Faculty of Medicine, Dentistry and Health Sciences, University of Melbourne, Melbourne, Australia; 3 Department of Cellular and Molecular Medicine, KU Leuven, Leuven, Belgium; 4 Department of Educational Studies, Faculty of Psychology and Educational Sciences, University of Ghent, Ghent, Belgium; 5 Health and Care Research, Artevelde University of Applied Sciences, Ghent, Belgium; 6 Center for General Practice, Department of Family Medicine and Population Health, University of Antwerp, Antwerp, Belgium

**Keywords:** Competency framework, CanMEDS, Delphi methodology, General practice, Postgraduate training, Workplace-based assessment

## Abstract

**Background:**

In view of the exponential use of the CanMEDS framework along with the lack of rigorous evidence about its applicability in workplace-based medical trainings, further exploring is necessary before accepting the framework as accurate and reliable competency outcomes for postgraduate medical trainings. Therefore, this study investigated whether the CanMEDS key competencies could be used, first, as outcome measures for assessing trainees’ competence in the workplace, and second, as consistent outcome measures across different training settings and phases in a postgraduate General Practitioner’s (GP) Training.

**Methods:**

In a three-round web-based Delphi study, a panel of experts (*n* = 25–43) was asked to rate on a 5-point Likert scale whether the CanMEDS key competencies were feasible for workplace-based assessment, and whether they could be consistently assessed across different training settings and phases. Comments on each CanMEDS key competency were encouraged. Descriptive statistics of the ratings were calculated, while content analysis was used to analyse panellists’ comments.

**Results:**

Out of twenty-seven CanMEDS key competencies, consensus was not reached on six competencies for feasibility of assessment in the workplace, and on eleven for consistency of assessment across training settings and phases. Regarding feasibility, three out of four key competencies under the role “Leader”, one out of two competencies under the role “Health Advocate”, one out of four competencies under the role “Scholar”, and one out of four competencies under the role “Professional” were deemed as not feasible for assessment in a workplace setting. Regarding consistency, consensus was not achieved for one out of five competencies under “Medical Expert”, two out of five competencies under “Communicator”,one out of three competencies under “Collaborator”, one out of two under “Health Advocate”, one out of four competencies under “Scholar”, one out of four competencies under “Professional”. No competency under the role “Leader” was deemed to be consistently assessed across training settings and phases.

**Conclusions:**

The findings indicate a mismatch between the initial intent of the CanMEDS framework and its applicability in the context of workplace-based assessment. Although the CanMEDS framework could offer starting points, further contextualization of the framework is required before implementing in workplace-based postgraduate medical trainings.

**Supplementary Information:**

The online version contains supplementary material available at 10.1186/s12909-023-04207-2.

## Background

In an era of greater accountability, the need for reassurance of medical competence among health professionals and trainees has revived the attention to competency-based medical education (CBME) [1]. This outcome-based approach provides fruitful ground for individual, programmatic, and institutional changes in order to align medical curricula with societal and patient expectations [2]. Implementation of CBME could offer guidance and direction to learners, and, at the same time, transparency and accountability to patients and to the general public. Following the International CBME Collaborators, competence designates an array of physicians’ abilities, whereas competency defines an observable and measurable physicians’ ability [1]. In CBME, predefined competencies and outcomes that go beyond medical knowledge and clinical reasoning are a prerequisite to meet high quality of patient care [3].

Medical educators have repeatedly attempted to define competencies for CBME. The result of these attempts is the creation of different competency frameworks for postgraduate medical education, like the Canadian Medical Education Directives for Specialists (CanMEDS) [4]. The CanMEDS competency framework has been originally developed by the Royal College of Physicians and Surgeons in Canada for defining educational outcomes for graduate medical education, and it is currently worldwide the most widely accepted and utilized framework within medical curricula [4, 5]. The CanMEDS framework identifies and describes different outcomes as competencies that physicians should acquire to follow patient-centred care. These competencies are thematically grouped under seven different roles: medical expert, communicator, collaborator, leader, health advocate, scholar, and professional [4]. The framework also splits competencies into two levels, level one contains the key competencies and level two the enabling competencies. The two levels together provide a multifaceted sum of descriptors to comprehend of what is expected by future physicians [6]. By encompassing various aspects of outcomes, the CanMEDS roles provide a comprehensive analytic framework for learning, teaching, and assessing in medical curricula [6].

In a workplace-based curriculum, the CanMEDS competencies could be captured through different workplace-based assessment tools [7]. To facilitate implementation of CBME in postgraduate medical training, changes in curricular structure are necessary. Among other curricular changes, CBME requires aligning learning outcomes with learning and assessment activities, and adopting learning outcomes that support educational continuity [8]. Consequently, as an outcome-based framework, the CanMEDS not only need to align with and accommodate the purposes of workplace-based assessment, but also to document and reflect competency growth across settings and time [3, 8].

Although context dependency and relevance have been demonstrated in the literature before, there is a lack of evidence about the extent to which the CanMEDS key competencies could be applied and implemented as accurate and reliable outcome measures in a workplace-based postgraduate medical training [9–12]. Therefore, this study aimed to investigate, firstly, whether the CanMEDS key competencies could be assessed in the workplace, and, subsequently, whether they could be consistently assessed across different training settings and phases as to document and reflect competency growth.

## Methods

We employed a web-based Delphi study to gather evidence based on consensus ratings on which CanMEDS key competencies had to be evaluated, first, as feasible, and, then as consistent for workplace-based assessments in the Flemish General Practitioner's (GP) Training, in Belgium [13–15]. Based on available literature, we discussed and decided on the necessary steps to ensure methodological rigor. Table 1 provides an overview of designing steps, based on the Guidance on Conducting and REportingDElphi Studies (CREDES) guideline [16]. We further elaborate on our methodological decisions considering the CREDES design steps.

**Table 1:** Steps for designing a Delphi study based on the CREDES guideline

• Defining the purpose of the Delphi study
• Definition of Delphi round
• Definition of (non) consensus
• Selection of expert panel
• Development and pilot of Delphi instrument
• Guidelines on interpreting results and proceeding between the rounds including informational input for experts
• Role of research team to prevent bias
• Strategies to improve response rate

### Study design

**Fig. 1:**
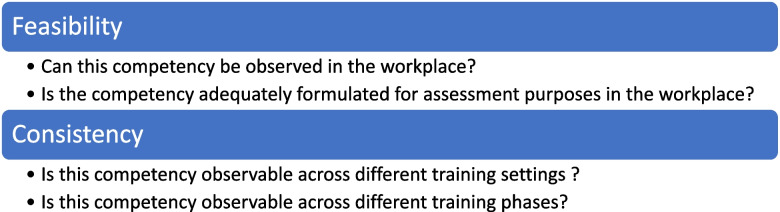
Definition of research criteria for the Delphi study

We chose to employ an e-Delphi to recruit panellists from diverse geographic locations within Flanders and to reach a larger group in a cost-efficient way. The online form was also preferred since this study took place during the COVID-19 pandemic. We defined feasibility as what can be observed in the workplace, and whether the competency formulation is suitable for workplace-based assessment. We defined consistency as what can be consistently observed across different training settings and phases in the workplace (Fig. 1) [13–15]. Consensus was defined as 70% of respondents agreed or strongly agreed that an item was feasible or consistent for assessment in the workplace [17]. Non-consensus was defined as less than 70% of respondents agreed or strongly agree, and no major change in consensus ratings nor any suggestions for change by the panel after 2 rounds.

To guarantee the reiterative nature of our study, we decided to set a minimum number of three rounds [18, 19]. After each Delphi round, when consensus was achieved for a CanMEDS key competency, the latter was no longer offered for evaluation. Although the traditional Delphi methodology commences with an unstructured round, we chose to follow a semi-structured approach, since our main goal was to validate the predefined CanMEDS framework [4]. Therefore, we used a combination of closed and open-ended questions [20].

In the first round, panellists were asked to evaluate the CanMEDS key competencies as feasible and as consistent based on a 5-point Likert scale. They were also able to provide qualitative comments for each key competency [7, 14]. In the second round, we informed the panellists about the consensus ratings of round 1. In this round, panellists were asked to formulate concrete suggestions for modifications, and rate the two research criteria separetely. A document was also added addressing the issues that arose in round 1 based on qualitative remarks. To provide some clarity about the formulation of the competencies, the CanMEDS, enabling competencies of each key competency were provided to assist the panel with their suggestions. Additionally, we listed and categorized the most frequent qualitative comments to provide an overview. Decisions about modifications on key competencies were clearly communicated. We asked the panel again to evaluate the CanMEDS key competencies as feasible and as consistent for workplace-based assessment based on 5-point Likert scale.

**Fig. 2:**
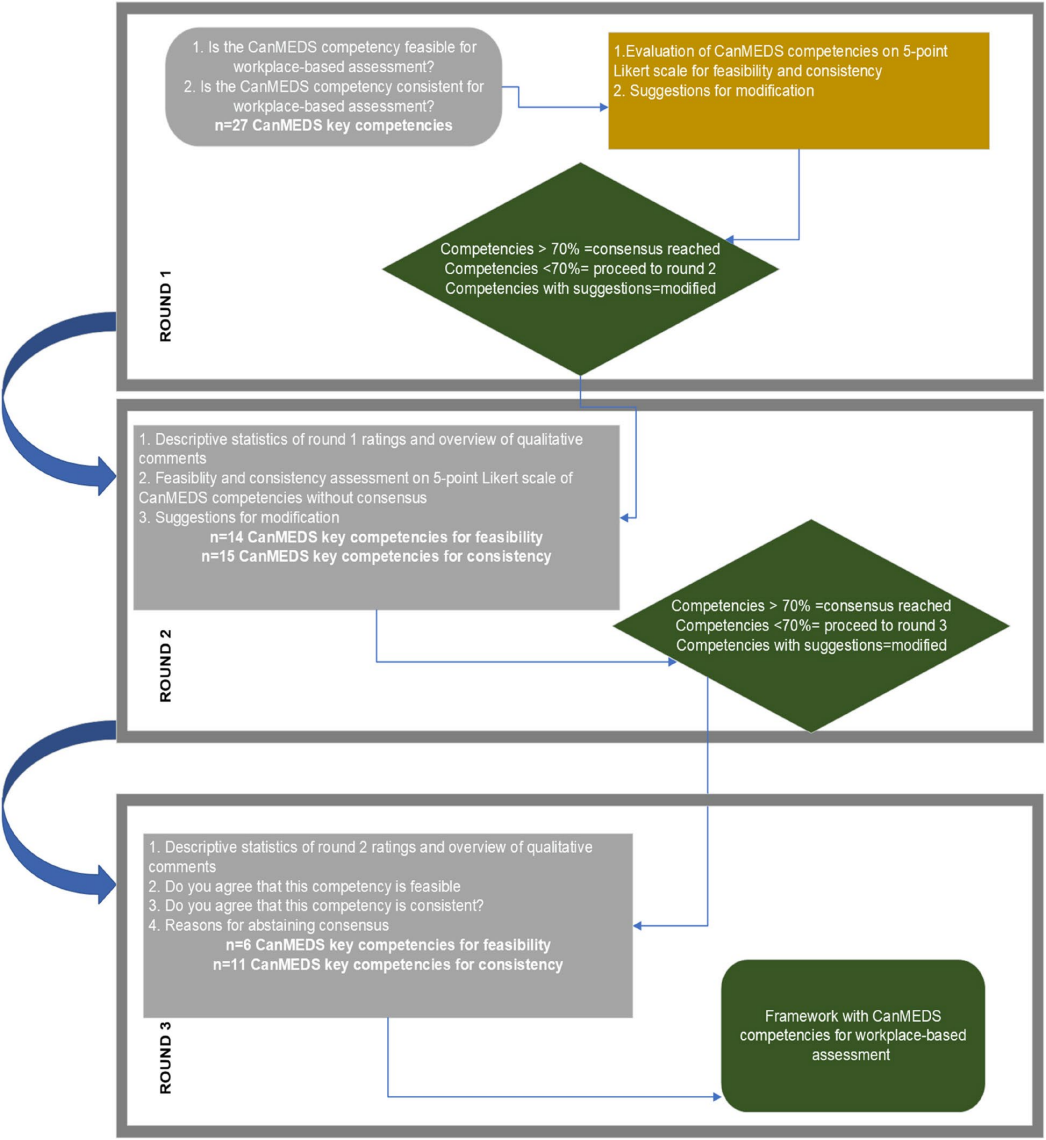
Flowchart of the three Delphi rounds

In the third round, we provided summaries of the ratings from the previous rounds. After panellists’ request, we included a list of examples of how each CanMEDS key competency would transfer to the workplace. In this final round, we asked the panellists whether they agree or not that a CanMEDS key competency was feasible and consistent for assessment in the workplace. If not, they were required to specify the reasons for abstaining consensus [15]. Figure 2 shows an overview of the three Delphi rounds.

### Study setting

To create a coherent approach across Flanders, four Flemish universities (KU Leuven, University of Ghent, University of Antwerp, and the Flemish Free University of Brussels) have created an interuniversity curriculum for the GP Training which consists of three phases. Practical coordination and decision-making regarding the curriculum are the responsibility of the Interuniversity Centre for GP Training (ICGPT). The ICGPT is responsible among others for allocating clinical internships, organizing exams, arranging fortnightly meetings of GP trainees with tutors, and handling trainees’ learning portfolios where evaluation of competencies is registered.

### Selection of panel

To select panellists, we followed a purposive sampling [13, 21]. We set three selection criteria: 1) having sufficient experience as a GP (> 3 years of experience), 2) having experience in mentoring and assessing trainees in the workplace, 3) having sufficient time and willingness to participate [7, 22]. Seventy panellists were invited by the Principal Investigator (BS) via email. To incorporate a wide range of opinions, the panel consisted of both GP trainers and GP tutors [23]. GP trainers were workplace-based trainers assisting trainees during their internship, while GP tutors were associated with a university providing guidance and facilitating peer learning and support (10–15 trainees per group) twice monthly. Both groups were responsible for assessing trainees in the workplace. Panellists resided in different provinces of Flanders to minimize converging ideas and to ensure reliability [13, 23]. Although there is no consensus about an appropriate sample size for a Delphi design, a number of 15–30 panellists could yield reliable results [23, 24]. In our study, we selected panellists that had received the same medical background and hold general understanding in the field of interest. In addition, to determine sample size, we took into consideration feasibility parameters to obtain a good response rate, such as providing large time spans for each Delphi round and reasonable required time to completion.

### Development and pilot of Delphi survey

**Fig. 3:**
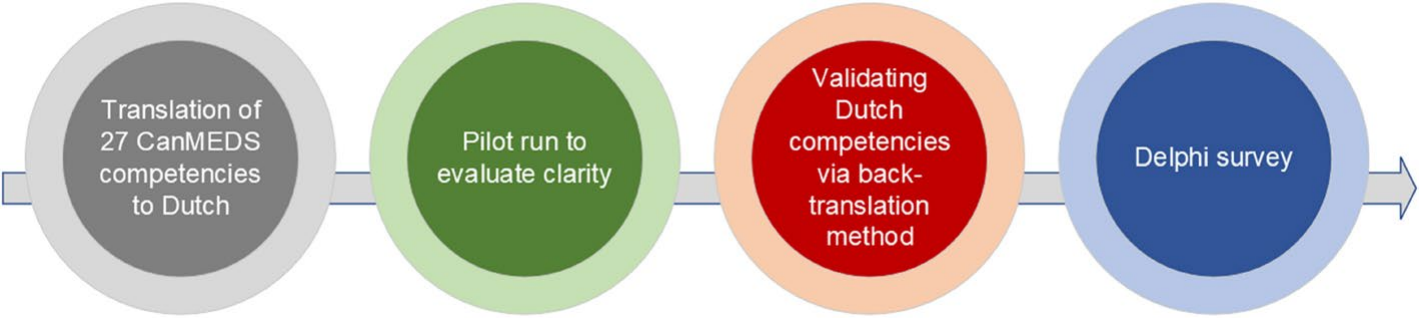
Process steps for constructing the Delphi survey

The 27 CanMEDS key competencies were translated from English to Dutch, because the panel was Dutch speaking. Figure 3 graphically illustrates how the Delphi survey was constructed. First, the CanMEDS competencies were translated by five researchers separately. After discussing and evaluating all translations, we decided to keep the Dutch translation as close as possible to the original English framework. Secondly, to validate the translation and pilot the instrument, we sent it to a group of medical professionals to comment on it. Thirdly, once feedback was received and the Dutch translation was finalized, the Dutch version of the framework was back translated to English to confirm the accuracy of the translation [25].

**Fig. 4:**
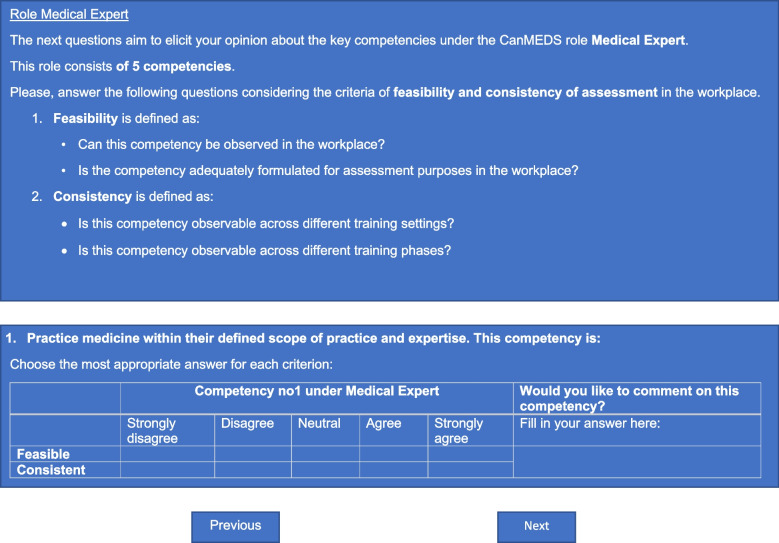
Display of survey items for Delphi round 1

Every Delphi round consisted of an introductory part, the CanMEDS key competencies evaluation, and an ending section. In the introduction, the purpose of each round was explained, and decision rules were communicated. We added the ending section to provide space to the panel for communication and feedback not related to the CanMEDS key competencies (e.g., necessary time to completion, remarks about layout). To avoid confusion among the different CanMEDS roles, the key competencies were grouped per role. Figure 4 illustrates how the survey items were displayed prior to any consensus had been reached.

### Data collection and analysis

 To collect our data, we used the Qualtrics XM Platform. This online tool allowed for maintaining anonymity among the panellists [26]. A personal link was sent via email to each panellist. This allowed following-up response rates and sending reminders to specific members. Due to high workload caused by the COVID-19 pandemic, each round lasted four weeks. We opted for a flexible approach towards the panellists to increase the response rate of each round. Reminders were weekly sent to members that had not completed the survey [26]. Data collection took place between October 2020 and February 2021. For analysing quantitative data, we calculated descriptive statistics of every item using SPSS 27 (IBM SPSS Statistics 27). We used Microsoft Excel to list and categorize qualitative data. Panellists’ comments were anonymously and literally registered. For analysing qualitative data, we used content analysis [27].

### Role of research team to prevent bias

Methodological decisions made by the research team were in line with the available literature. We predefined and stipulated methodological steps before commencing the study. We applied, monitored, and evaluated these steps during the study. The results of each round were discussed by the research team, while qualitative data were interpreted by two researchers for researcher triangulation [28].

## Results

Initially, 53 GP trainers and GP tutors chose to participate in the panel. Forty-three out of 53 responded in the first Delphi round (response rate of 89.4%) (Table 2). Twenty-five out of 43 were female, while 18 were male (Table 3). Most panellists (*n* = 27) were 36 years old or older and mainly GP trainers (*n* = 31) with at least 5 years of experience (*n* = 27) in guiding GP trainees in the workplace, and at least 5 years of experience (*n* = 26) in assessing GP trainees in the workplace. The second Delphi round had a response rate of 88.9% (*n* = 33), while the third round had a response rate of 76.12% (*n* = 24) (Table 2). Panellists who chose to withdraw their participation attributed this to the COVID-19 related increase of workload in their clinical practice. Tables 4 and 5 provide an overview of the level of consensus per Delphi round for feasibility and consistency of assessment respectively. An additional file presents the number of comments on feasibility and consistency of assessment per CanMEDS key competency, per role, and per Delphi round (see Additional file 1).

**Table 2:** Response rates per Delphi round

Delphi round	N	Response rate
round 1	43	89.4%
round 2	33	88.9%
round 3	25	76.12%

**Table 3:** Composition of panel

	**N**
Number of participants in round 1	43
**Sex**	
Male	18
Female	25
**Age**	
25–35 years old	4
36–45 years old	12
46–55 years old	14
56 years old or older	13
**Role in the GP Training**	
GP trainer	31
GP tutor	6
Both	6
**Flemish region**	
West Flanders	7
East Flanders	13
Brussels	2
Antwerp	10
Flemish Brabant	6
Limburg	5
**Years as GP**	
Between 3 and 5 years	1
Between 6 and 10 years	6
More than 10 years	36
**Years of experience in guiding GP trainees**	
Less than 3 years	4
Between 3 and 5 years	12
Between 6 and 10 years	12
More than 10 years	15
**Years of experience in assessing GP trainees in the workplace**	
Less than 3 years	5
Between 3 and 5 years	13
Between 6 and 10 years	11
More 10 years	14

**Table 4:** Consensus ratings on feasibility of the CanMEDS competencies per Delphi round

CanMEDS Roles	CanMEDS competencies	Level of consensus: Is this CanMEDS competence feasible for assessment in the workplace?		
		**Round 1**	**Round 2**	**Round 3**
MEDICAL EXPERT The GP trainee is able to:	1. Practice medicine within their defined scope of practice and expertise	**71.4%**		
	2. Perform a patient-centred clinical assessment and establish a management plan	**71.4%**		
	3. Plan and perform procedures and therapies for the purpose of assessment and/or management	**71.4%**		
	4. Establish plans for ongoing care and, when appropriate, timely consultation	57.1%	**100%**	
	5. Actively contribute, as an individual and as a member of a team providing care, to the continuous improvement of health care quality and patient safety	40%	56%	**72.2%**
COMMUNICATOR The GP trainee is able to:	1. Establish professional therapeutic relationships with patients and their families	38.2%	**87.5%**	
	2. Elicit and synthesize accurate and relevant information, incorporating the perspectives of patients and their families	51.5%	66.7%	**83.3%**
	3. Share health care information and plans with patients and their families	53%	**91.7%**	
	4. Engage patients and their families in developing plans that reflect the patient’s health care needs and goals	41.2%	52.1%	**77.8%**
	5. Document and share written and electronic information about the medical encounter to optimize clinical decision-making, patient safety, confidentiality, and privacy	54.3%	**79.1%**	
COLLABORATOR The GP trainee is able to:	1. Work effectively with physicians and other colleagues in the health care professions	45.7%	**87.5%**	
	2. Work with physicians and other colleagues in the health care professions to promote understanding, manage differences, and resolve conflicts	34.3%	54.2%	**88.9%**
	3. Hand over the care of a patient to another health care professional to facilitate continuity of safe patient care	48.5%	62.5%	**83.4%**
LEADER The GP trainee is able to:	1. Contribute to the improvement of health care delivery in teams, organizations, and systems	34.3%	45.9%	55.6%
	2. Engage in the stewardship of health care resources	33.9%	50%	**72.2%**
	3. Demonstrate leadership in professional practice	42.8%	50%	50%
	4. Manage career planning, finances, and health human resources in a practice	28.6%	25%	50%
HEALTH ADVOCATE The GP trainee is able to:	1. Respond to an individual patient’s health needs by advocating with the patient within and beyond the clinical environment	38.9%	**70.8%**	
	2. Respond to the needs of the communities or populations they serve by advocating with them for system-level change in a socially accountable manner	16.7%	25%	44.5%
SCHOLAR The GP trainee is able to:	1. Engage in the continuous enhancement of their professional activities through ongoing learning	**80.6%**		
	2. Teach students, residents, the public, and other health care professionals	36.1%	54.2%	61.1%
	3. Integrate best available evidence into practice	**77.7%**		
	4. Contribute to the creation and dissemination of knowledge and practices applicable to health	52.7%	58.30%	**77.8%**
PROFESSIONAL The GP trainee is able to:	1. Demonstrate a commitment to patients by applying best practices and adhering to high ethical standards	63.9%	**70.9%**	
	2. Demonstrate a commitment to society by recognizing and responding to societal expectations in health care	31.5%	33.3%	44.5%
	3. Demonstrate a commitment to the profession by adhering to standards and participating in physician-led regulation	50%	**78.2%**	
	4. Demonstrate a commitment to physician health and well-being to foster optimal patient care	38.9%	43.5%	**88.9%**

**Table 5:** Consensus ratings on consistency of the CanMEDS competencies per Delphi round

CanMEDS Roles	CanMEDS competencies	Level of consensus: Is this CanMEDS competence consistent for assessment in the workplace?		
		**Round 1**	**Round 2**	**Round 3**
MEDICAL EXPERT The GP trainee is able to:	1. Practice medicine within their defined scope of practice and expertise	65.7%	**84%**	
	2. Perform a patient-centred clinical assessment and establish a management plan	**71.4%**		
	3. Plan and perform procedures and therapies for the purpose of assessment and/or management	**77.2%**		
	4. Establish plans for ongoing care and, when appropriate, timely consultation	54.3%	**88%**	
	5. Actively contribute, as an individual and as a member of a team providing care, to the continuous improvement of health care quality and patient safety	48.5%	60%	55.6%
COMMUNICATOR The GP trainee is able to:	1. Establish professional therapeutic relationships with patients and their families	51.5%	**75%**	
	2. Elicit and synthesize accurate and relevant information, incorporating the perspectives of patients and their families	57.6%	50%	66.7%
	3. Share health care information and plans with patients and their families	52.9%	**79.2%**	
	4. Engage patients and their families in developing plans that reflect the patient’s health care needs and goals	41.2%	39.1%	44.4%
	5. Document and share written and electronic information about the medical encounter to optimize clinical decision-making, patient safety, confidentiality, and privacy	54.2%	**79.2%**	
COLLABORATOR The GP trainee is able to:	1. Work effectively with physicians and other colleagues in the health care professions	51.4%	**87.5%**	
	2. Work with physicians and other colleagues in the health care professions to promote understanding, manage differences, and resolve conflicts	40%	54.1%	66.7%
	3. Hand over the care of a patient to another health care professional to facilitate continuity of safe patient care	54.3%	62.5%	**83.3%**
LEADER The GP trainee is able to:	1. Contribute to the improvement of health care delivery in teams, organizations, and systems	42.8%	37.5%	55.6%
	2. Engage in the stewardship of health care resources	37.2%	45.8%	66.6%
	3. Demonstrate leadership in professional practice	45.7%	54.2%	54.2%
	4. Manage career planning, finances, and health human resources in a practice	48.6%	20.9%	50%
HEALTH ADVOCATE The GP trainee is able to:	1. Respond to an individual patient’s health needs by advocating with the patient within and beyond the clinical environment	42.9%	58.3%	**77.8%**
	2. Respond to the needs of the communities or populations they serve by advocating with them for system-level change in a socially accountable manner	13.9%	25%	38.9%
SCHOLAR The GP trainee is able to:	1. Engage in the continuous enhancement of their professional activities through ongoing learning	**77.7%**		
	2. Teach students, residents, the public, and other health care professionals	36.1%	50%	**72.2%**
	3. Integrate best available evidence into practice	**83.4%**		
	4. Contribute to the creation and dissemination of knowledge and practices applicable to health	47.2%	54.1%	61.1%
PROFESSIONAL The GP trainee is able to:	1. Demonstrate a commitment to patients by applying best practices and adhering to high ethical standards	**72.2%**		
	2. Demonstrate a commitment to society by recognizing and responding to societal expectations in health care	28.6%	30.4%	44.5%
	3. Demonstrate a commitment to the profession by adhering to standards and participating in physician-led regulation	52.8%	**78.2%**	
	4. Demonstrate a commitment to physician health and well-being to foster optimal patient care	38.8%	43.5%	**83.4%**

### Delphi round 1

In the first Delphi round, five out of 27 CanMEDS competencies reached a positive 70% consensus rate for feasibility and 3 competencies for consistency (Tables 4 and 5 respectively). In total, the panellists gave 154 qualitative comments, 130 comment about feasibility and 24 about consistency of assessment. Those comments were clustered into six main categories: 1. CanMEDS key competency not suitable for workplace-based assessment via a portfolio (*n* = 46), 2. Vague formulation of CanMEDS key competency (*n* = 19), 3. Assessment of CanMEDS key competency dependent on phase in GP Training (*n* = 16), 4. CanMEDS key competency not applicable during GP Training (*n* = 15), 5. Assessment of some CanMEDS key competencies dependent on location of clinical practice and patient population (*n* = 12), and 6. Assessment of CanMEDS key competency dependent on trainer and/or trainee (*n* = 12).

### Delphi round 2

In the second Delphi round, the panel was asked to rate 22 CanMEDS key competencies for feasibility and 23 for consistency of assessment in the workplace. As seen in Table 4 and in Table 5, the panel reached consensus for 8 out of 22 CanMEDS key competencies for feasibility and for 8 out of 23 for consistency of assessment. Overall, the consensus rates increased. However, a key competency (n°4) under the role “Leader” had lower scores on both feasibility and consistency comparing to the ones from round one (Tables 4 and 5). Additionally, key competency n°1 under “Leader” scored lower on consistency than in round 1, while consistency consensus rates on key competency n°2 under “Communicator” also decreased in round 2. No adjustments were made to the formulation since no panellists’ comments suggested any alterations.

In total, 117 remarks were given by the panel that were listed in four main clusters: 1. Vague formulation (*n* = 48), 2. CanMEDS key competency not applicable in GP Training (*n* = 13), 3. Suggestions for operationalization (*n* = 14), and 4. CanMEDS key competency could only be assessed by GP trainer (*n* = 10). Two comments were about the fact that assessment of some key competencies was dependent on the location of clinical practice or the context, one comment was about dependency of assessment on training phase, while two comments suggested some overlap between key competencies n°3 and n°4 under the “Medical Expert” role. Fifteen comments were deemed as not relevant and, therefore, categorized as others.

At this point, despite the lack of concrete suggestions, low consensus levels on the key competencies under the “Leader” role led to modifying three out of four key competencies. More modifications were made to key competency n°2 under “Health Advocate” to make it more comprehensible, while key competency n°2 under “Scholar” was also modified to emphasize the importance of educating colleagues in the same discipline. Additional file 2 illustrates in detail the CanMEDS competencies that were modified (see Additional file 2). To ensure that the modifications aligned with the CanMEDS framework, we ensured that the modified competencies contained the same key words and kept the same focus as the original CanMEDS competencies. These modifications were based on all the data after two Delphi rounds and after discussions within the research team.

### Delphi round 3

The third Delphi round included 12 CanMEDS key competencies to be rated for feasibility of assessment, and 15 to be rated for consistency of assessment in the workplace. In this round, 6 out of 12 competencies were rated as feasible, while 4 out 15 were rated as consistent. Panellists made 39 comments about the importance of the CanMEDS competencies for the workplace and suggestions about the way of assessing them in the clinical setting. Panellists did not agree on 6 CanMEDS key competencies about feasibility and on 11 key competencies about consistency. No further round deemed necessary since panellists’ comments did not provide any insights for further modifications.

## Discussion

The aim of this study was to collect evidence about applying and implementing the CanMEDS competency famework for workplace-based assessment by employing a Delphi study. The CanMEDS competency framework is well-known globally and has increasingly been incorporated in postgraduate medical education [6]. A competency framework can facilitate the development of competencies, albeit it is not the primary goal. Although an outcome-based approach would not lead to learning itself, it undoubtfully establishes all the conditions leading to learning. Implementing competency frameworks prerequisites defining a clear path of the desired learning outcomes for the learners, providing occasions to exercise these outcomes within and across settings, creating opportunities for assessment and feedback, and enhancing reflection on individual performance [29]. Subsequently, a developmental learning trajectory and competency growth are presumably inherent components of competency frameworks.

However, applicability of the CanMEDS framework seems to be dependent on the context and on medical specialty, while evidence about how competency growth can be documented and reflected through the framework is scarce [9, 30, 31]. This study focused on two criteria, feasibility of assessment and consistency of assessment across training settings and phases, embedded in a workplace-based postgraduate GP Training. Our findings show issues related to the fitness-for- purpose of the CanMEDS key competencies, and to the extent that they can be used for assessment purposes throughout the course of a workplace-based postgraduate training.

Regarding the assessment feasibility, our research indicates that not all CanMEDS key competencies could be clearly related to observable behaviour. Some CanMEDS key competencies under the “Leader”, “Health Advocate”, and “Professional” role got notably lower scores (< 50%) compared to competencies under other CanMEDS roles, such as “Medical Expert”, “Communicator”, and “Scholar”. The big discrepancies in the rating scores might imply that the panel experienced difficulties in associating how those CanMEDS competencies could be transferred and translated into assessment activities in the workplace. This is in line with previous research showing difficulties in applying and using the roles of “Health Advocate and “Leader” in undergraduate medical curricula [7]. Difficulty in applying the CanMEDS key competencies in workplace-based assessment has been attributed to the lack of coherent and concrete descriptions, in undergraduate medical education [7]. In postgraduate medical education, difficulty in implementing the CanMEDS non-medical competencies has been associated with the lack of training for workplace trainers [32]. In our study, panellists also seemed to be more familiar with competencies focusing on medical knowledge, clinical and communication skills rather than with competencies involving societal and public engagement. Clearly, the predominance of clinical and communication skills reflects the role of a GP in primary health [33]. However, medical educators should consider that public education and engagement is a prerequisite for a more equitable and patient-centred healthcare system [34].

Our exploration of consistency across training settings and phases of the CanMEDS key competencies yielded unanticipated results as well. Similar patterns as in the ratings of assessment feasibility were also found in those regarding assessment consistency of the CanMEDS key competencies. Strikingly, almost half of the CanMEDS key competencies seemed unsuitable for consistently being observed in the workplace across different training settings and phases. Remarkably, none of the key competencies under the “Leader” role was deemed as consistent. Presumably, the panel may have reported consistency based on their role in the GP Training. They seemed to pay attention to under which conditions (e.g., comments about “Practice/context dependent”) and by whom (e.g., comments about assesed “only by trainer”) each competency could be assessed. Our results suggest a potential mismatch between the CanMEDS key competencies and certain training settings and phases. Evidence in existing literature also shows that medical students associate the least the roles of “Leader”, “Health Advocate”, “Collaborator”, and “Professional” to their learning activities [35]. This problematic might indicate a dissociation between the CanMEDS framework and its ability to document and reflect competency growth across different training settings and phases.

Our findings suggest that there is a gap between the initial intent of the CanMEDS competency framework and its applicability when it comes to workplace-based assessment [12]. Although the importance of the framework was reinstated by the panel, the difficulty of reaching consensus throughout the three Delphi rounds elucidates issues regarding its implementation in the clinical workplace. The CanMEDS key competencies might offer a starting point for structuring workplace-based assessment and capturing medical competence. Nevertheless, further refinement and contextualisation of the framework is necessary to assist observations of trainees’ behaviour involving all seven CanMEDS roles during clinical practice. Future research should explore implementation issues in different health care contexts and settings to gather more evidence on the CanMEDS framework.

### Limitations

We acknowledge several limitations of this Delphi study. Notably the fact that the CanMEDS framework was translated in Dutch might have caused some degree of linguistic bias. Since language arguably carries cultural associations and meanings, a translation of the original framework could presumably miss cultural elements inherent to the Flemish GP Training. However, choosing to stay as close to the original framework as possible implies that our findings could be generalized and used in other international contexts. Furthermore, although we provided explicit instructions on how to fill in each round, we cannot exclude that some panellists might have been confused about how to respond to the set of our research questions. Another limitation that needs to be acknowledged is the high dropout rate, since 43 panellists had initially participated in round 1. Unfortunately, the beginning of our study coincided with the onset of a COVID-19 wave. Consequently, the increased workload in clinical practice did not allow some panellists to further participate in this study. A final limitation is that the Delphi method is merely a consensus method based on experts’ opinion. The panel might have answered our research questions having their own preconceptions and interpretations of the CanMEDS framework, for example, how they understand its applicability and implementation in the clinical workplace. Admittedly, this limitation is inherent in every opinion-based method. Nevertheless, by comprehensively describing and justifying every methodological choice, it can be sufficiently argued that our findings are credible and useful.

## Conclusions

This study aimed to gather evidence on the applicability and implementation of the CanMEDS key competencies for workplace-based assessment purposes. Given that CBME is increasingly implemented across the globe, the findings provide some insight into the implementation of the CanMEDS framework and its fitness-for-purpose for assessment in the workplace. Drawing on the results of this Delphi study, adapting and adopting the current CanMEDS key competencies should be considered before implementation in postgraduate medical trainings. The challenge for medical educators relates to how to encompass and capture in a workplace-based learning environment aspects of medical competence besides medical knowledge, and clinical and communication skills. Lastly, for CBME to pertain, more attention should be paid to how the CanMEDS competencies could be used as educational outcomes across different training settings and phases. There is a need for rigorous evidence on how the CanMEDS framework can document and reflect competency growth in the workplace.

## Supplementary Information

**Additional file 1:** **Table 6.** Number of panellists’ comments per CanMEDS competency and research criterion in every Delphi round.

**Additional file 2:** **Table 7.** Overview of modified CanMEDS key competencies.

## Data Availability

The datasets used and analysed during the current study are available from the corresponding author on reasonable request.

## Abbreviations

GP: General Practitioner
CBME: Competency-based medical education
CanMEDS: Canadian Medical Education Directives for Specialists
CREDES: Guidance on Conducting and REportingDElphi Studies guideline
ICGPT: Interuniversity Centre for GP Training
