# Supplementary material for: Fitness-for-purpose of the CanMEDS competencies for workplace-based assessment in General Practitioner’s Training: a Delphi study
Source: BMC Med Educ. 2023 Apr 1;23:204. doi: 10.1186/s12909-023-04207-2 (PMC10067520; doi:10.1186/s12909-023-04207-2)
Supplement: Supplementary file 1 — Additional file 1: Table 6. Number of panellists’ comments per CanMEDS competency and research criterion in every Delphi round. [file 12909_2023_4207_MOESM1_ESM.docx]

Table 6 Number of panellists’ comments per CanMEDS competency and research criterion in every Delphi round

| **CanMEDS roles** | **CanMEDS competencies** |  | **Number of comments** | | | | | | | | | |  |
| --- | --- | --- | --- | --- | --- | --- | --- | --- | --- | --- | --- | --- | --- |
|  |  |  | *Round 1 (n=156)* | | | *Round 2 (n=117)* | | | *Round 3 (n=39)* | | | |  |
|  |  |  | ***Per competency*** | ***Per research criterion*** | | ***Per competency*** | ***Per research criterion*** | | ***Per competency*** | ***Per research criterion*** | | |  |
|  |  |  |  | Feasibility | Consistency |  | Feasibility | Consistency |  | | Feasibility | Consistency | |
| MEDICAL EXPERT The GP trainee is able to: | 1. Practice medicine within their defined scope of practice and expertise. |  | 7 | 6 | 1 | 4 | **V** | 4 |  | | **V** | **V** | |
|  | 2. Perform a patient-centred clinical assessment and establish a management plan. |  | 5 | 4 | 1 |  | **V** | **V** |  | | **V** | **V** | |
|  | 3. Plan and perform procedures and therapies for the purpose of assessment and/or management. |  | 3 | 3 |  |  | **V** | **V** |  | | **V** | **V** | |
|  | 4. Establish plans for ongoing care and, when appropriate, timely consultation. |  | 5 | 5 |  | 6 | 4 | 2 |  | | **V** | **V** | |
|  | 5. Actively contribute, as an individual and as a member of a team providing care, to the continuous improvement of health care quality and patient safety. |  | 5 | 4 | 1 | 4 | 3 | 3 | 5 | | 3 | 2 | |
|  | **Total N comments MEDICAL EXPERT** | **25** | | | | **13** | | | **5** | | | |  |
| COMMUNICATOR The GP trainee is able to: | 1. Establish professional therapeutic relationships with patients and their families. |  | 9 | 9 |  | 10 | 5 | 5 |  | | **V** | **V** | |
|  | 2. Elicit and synthesize accurate and relevant information, incorporating the perspectives of patients and their families. |  | 6 | 6 | 6 | 9 | 3 | 6 | 4 | | 2 | 2 | |
|  | 3. Share health care information and plans with patients and their families. |  | 2 | 1 | 1 | 6 | 4 | 2 |  | | **V** | **V** | |
|  | 4. Engage patients and their families in developing plans that reflect the patient’s health care needs and goals. |  | 5 | 4 | 1 | 7 | 3 | 4 | 2 | | 2 |  | |
|  | 5. Document and share written and electronic information about the medical encounter to optimize clinical decision-making, patient safety, confidentiality, and privacy. |  | 4 | 3 | 1 | 7 | 4 | 3 |  | | **V** | **V** | |
|  | **Total n comments COMMUNICATOR** | **26** | | | | **39** | | | **6** | | | |  |
| COLLABORATOR The GP trainee is able to: | 1. Work effectively with physicians and other colleagues in the health care professions. |  | 10 | 7 | 3 | 2 | 2 |  |  | | **V** | **V** | |
|  | 2. Work with physicians and other colleagues in the health care professions to promote understanding, manage differences, and resolve conflicts. |  | 5 | 5 |  | 1 | 1 |  | 1 | | 1 |  | |
|  | 3. Hand over the care of a patient to another health care professional to facilitate continuity of safe patient care. |  | 5 | 4 | 1 | 4 | 4 |  | 2 | | 1 | 1 | |
|  | **Total N comments COLLABORATOR** | **20** | | | | **7** | | | **3** | | | |  |
| LEADER The GP trainee is able to: | 1. Contribute to the improvement of health care delivery in teams, organizations, and systems. |  | 10 | 8 | 2 | 5 | 3 | 2 | 4 | | 4 |  | |
|  | 2. Engage in the stewardship of health care resources. |  | 10 | 8 | 2 | 5 | 3 | 2 | 3 | | 3 |  | |
|  | 3. Demonstrate leadership in professional practice. |  | 8 | 5 | 3 | 5 | 3 | 2 | 5 | | 4 | 1 | |
|  | 4. Manage career planning, finances, and health human resources in a practice. |  | 7 | 6 | 1 | 8 | 4 | 4 | 1 | | 1 |  | |
|  | **Total N comments LEADER** | **35** | | | | **23** | | | **13** | | | |  |
| HEALTH ADVOCATE The GP trainee is able to: | 1. Respond to an individual patient’s health needs by advocating with the patient within and beyond the clinical environment. |  | 7 | 5 | 2 | 5 | 3 | 2 | 2 | | **V** | 2 | |
|  | 2. Respond to the needs of the communities or populations they serve by advocating with them for system-level change in a socially accountable manner. |  | 7 | 6 | 1 | 6 | 4 | 2 | 3 | | 3 |  | |
|  | **Total N comments HEALTH ADVOCATE** | **14** | | | | **11** | | | **5** | | | |  |
| SCHOLAR The GP trainee is able to: | 1. Engage in the continuous enhancement of their professional activities through ongoing learning. |  | 5 | 4 | 1 |  | **V** | **V** |  | | **V** | **V** | |
|  | 2. Teach students, residents, the public, and other health care professionals. |  | 8 | 6 | 2 | 5 | 4 | 1 | 3 | | 3 |  | |
|  | 3. Integrate best available evidence into practice. |  | 3 | 3 |  |  | **V** | **V** |  | | **V** | **V** | |
|  | 4. Contribute to the creation and dissemination of knowledge and practices applicable to health. |  | 5 | 5 |  | 4 | 3 | 1 | 3 | | 2 | 1 | |
|  | **Total N comments SCHOLAR** | **22** | | | | **9** | | | **6** | | | |  |
| PROFESSIONAL The GP trainee is able to: | 1. Demonstrate a commitment to patients by applying best practices and adhering to high ethical standards. |  | 3 | 3 |  | 2 | 2 | **V** |  | | **V** | **V** | |
|  | 2. Demonstrate a commitment to society by recognizing and responding to societal expectations in health care. |  | 3 | 3 |  | 5 | 5 |  | 1 | | 1 |  | |
|  | 3. Demonstrate a commitment to the profession by adhering to standards and participating in physician-led regulation. |  | 2 | 2 |  | 2 | 1 | 1 |  | | **V** | **V** | |
|  | 4. Demonstrate a commitment to physician health and well-being to foster optimal patient care. |  | 6 | 5 | 1 | 5 | 4 | 1 |  | |  |  | |
|  | **Total N comments PROFESSIONAL** | **14** | | | | **15** | | | **1** | | | |  |

(V=validated in previous round)
