# Supplementary material for: Fitness-for-purpose of the CanMEDS competencies for workplace-based assessment in General Practitioner’s Training: a Delphi study
Source: BMC Med Educ. 2023 Apr 1;23:204. doi: 10.1186/s12909-023-04207-2 (PMC10067520; doi:10.1186/s12909-023-04207-2)
Supplement: Supplementary file 2 — Additional file 2: Table 7. Overview of modified CanMEDS key competencies. [file 12909_2023_4207_MOESM2_ESM.docx]

Table 7 Overview of adjusted CanMEDS key competencies

| **CanMEDS role** | **Original CanMEDS key competency** | **Modified CanMEDS key competency** |
| --- | --- | --- |
| LEADER  The GP trainee is able to: | 1. Contribute to the improvement of health care delivery in teams, organizations, and systems. | 1. Contribute to the optimalization of quality of care in teams, organization, and systems. |
|  | 2.Engage in the stewardship of health care resources. | 2. Contribute actively to the efficient use of health care resources. |
|  | 4. Manage career planning, finances, and health human resources in practice. | 4. Manage career planning, take into account finances, and manages human resources when applicable. |
| HEALTH ADVOCATE  The GP trainee is able to: | 2. Respond to the needs of the communities or populations they serve by advocating with them for system-level change in a socially accountable manner. | 2. Promote the health of the communities and populations by contributing to systemic changes (lifestyle, social, and/or financial) in a socially responsible way. |
| SCHOLAR  The GP trainee is able to: | 2. Teach students, residents, the public, and other health care professionals. | 2. Teach students, trainees, colleagues from their own and other disciplines, and the public. |
